# A case of tracheal obstruction caused by reflux and aspiration of semi-solid nutrients via the nasogastric tube

**DOI:** 10.1016/j.ijscr.2019.11.004

**Published:** 2019-11-08

**Authors:** Masatoshi Nakagawa, Kaori Sugihara, Kiyoshi Isobe, Masafumi Akasu, Kazutaka Tsujimoto, Yasuhiro Itsui, Yasuaki Nakajima

**Affiliations:** a Department of Gastrointestinal Surgery, Tokyo Medical and Dental University, Japan; b Department of Clinical Nutrition, Tokyo medical and Dental University, Japan; c Department of Nephrology, Tokyo Medical and Dental University, Japan; d Department of Hepato-Biliary-Pancreatic Surgery, Tokyo Medical and Dental University, Japan; e Department of Molecular Endocrinology and Metabolism, Tokyo Medical and Dental University, Japan; f Department of Gastroenterology and Hepatology, Tokyo Medical and Dental University, Japan

**Keywords:** Semi-solid nutrients, Nasogastric tube, Enteral feeding, Tracheal obstruction, Case report

## Abstract

**Introduction:**

Semi-solid nutrients have several advantages, including reduced cases of diarrhea and aspiration pneumonia, and are usually administered via percutaneous endoscopic gastrostomy owing to its high viscosity. Administering semi-solid nutrients via a nasogastric tube was recently introduced in clinical practice; however, its safety has not been well confirmed.

**Presentation of case:**

An 82-year-old man with a right occipital hemorrhage and severe diarrhea consulted the nutritional support team. Administrations of semi-solid nutrients (HINE E-GEL®) via the nasogastric tube was initiated, which gradually alleviated his symptoms. Fourteen days after initiation, he suddenly had pulmonary failure owing to a tracheal obstruction caused by the reflux and aspiration of semi-solid nutrients. Intubation and subsequent reflex cough expectorated sputum with gel-form particles, which quickly stabilized his pulmonary condition. After this, his hospital course was stable, and he was referred to another hospital for further rehabilitation.

**Discussion:**

Semi-solid nutrients administered via the nasogastric tube have different ingredients compared with those administered via percutaneous endoscopic gastrostomy. HINE E-GEL®, for example, contains pectin and calcium phosphate that changes from liquid to semi-solid inside the stomach via chemical reactions under acidic conditions. Data on the viscosity of HINE E-GEL® *in vivo* are insufficient. Uncertainty regarding the form and viscosity of HINE E-GEL® inside the stomach complicates clinical practice.

**Conclusions:**

Although semi-solid nutrients have several advantages, including reduced diarrhea and gastroesophageal reflux, evidence on semi-solid nutrients via the nasogastric tube is insufficient. It should be noted that semi-solid nutrient reflux can be more fatal than liquid nutrients.

## Highlights

• There are not sufficient data on viscosity of semi-solid nutrients via the nasogastric tube in vivo.
• Tracheal obstruction occurred by reflux and aspiration of semi-solid nutrients through the nasogastric tube
• Reflux of semi-solid nutrients is rare, but once it occurs, it can be fatal.

## 1 Introduction

Semi-solid nutrients have a unique characteristic of slow movement in the stomach due to its high viscosity. They are useful for patients with diarrhea or reflux from enteral feeding. There are some reports insisting the safety and benefits of semi-solid nutrients such as reduced diarrhea, shorter administration time, and lower frequency of aspiration pneumonia [[1], [2], [3]]. Semi-solid nutrients are usually given through percutaneous endoscopic gastrostomy (PEG), but it is unsuitable for nasogastric tube administration due to its high viscosity. Recently, semi-solid nutrients for the nasogastric tube were introduced and frequently used in clinical practice [[4], [5], [6], [7]]. However, there is no sufficient evidence regarding the safety of semi-solid nutrients through the nasogastric tube. Here we present a patient who experienced tracheal obstruction caused by the reflux of semi-solid nutrients through the nasogastric tube, and subsequent pulmonary failure. The work has been reported in line with the SCARE 2018 Statement [8].

## 2 Presentation of case

### 2.1 History of present illness and past medical history

An 82-year-old man found unconscious at home was admitted to our hospital. He was subsequently diagnosed with a right occipital cerebral hemorrhage and perforation of the cerebral ventricle. He underwent emergency craniotomy for hematoma drainage. Enteral nutrition with semi-digestive nutrients through the nasogastric tube was started on postoperative day (POD) 2. The total dose and speed of administration were gradually increased, and patient started to have diarrhea. The speed of administration was reduced, which partially alleviated his symptoms. He also underwent dermatoplasty for decubitus on the left forearm, which already existed at the time of emergency admission, on POD 20. He was referred to the nutritional support team (NST) on POD 23 for further assessment and nutritional planning. His past medical history included colon cancer, cerebral infarction, hypertension, and prostate hypertrophy.

### 2.2 Physical examination and laboratory data

His physical findings at the time of consultation were as follows: conscious level (Glasgow coma Scale) of E2V3M5, left hemiplegia, bedridden status, Bristol stool scale of 6–7, frequency of defecation (3–8 times/day), height (170 cm), body weight (54.1 kg), and body mass index (18.7/kg/m^2^).

Abnormal findings and nutritional indices in laboratory test results were as follows:hemoglobin, 8.0 g/dL; albumin, 1.8 g/dL; potassium, 3.3 mEq/L; aspartate transaminase, 52 U/L; aspartate alanine transaminase, 63 U/L; total cholesterol, 108 mg/dL; C-reactive protein, 6.66 mg/dl; glucose, 184 mg/dl; copper, 64 μg/dL, and zinc, 63 μg/dL.

### 2.3 Hospital course after NST consultation

**Fig. 1:**
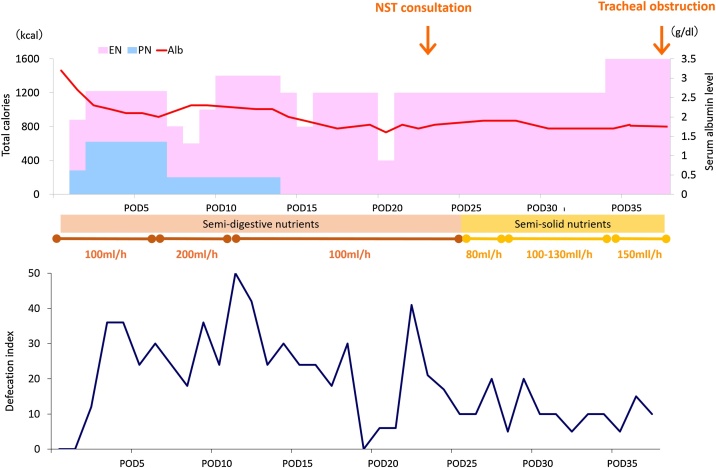
Hospital course of the patient. Defecation index was calculated as defecation times per day multiplied by the Bristol stool scale. EN: enteral nutrition; PN: peripheral nutrition; NST: nutrition support team; POD: postoperative day.

**Fig. 2:**
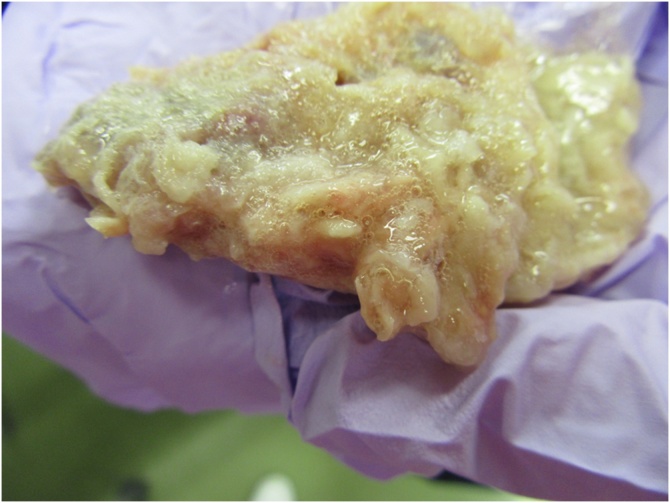
Pale yellow gel form which was expectorated from the mouth (7 × 5 × 3 cm).

Hospital course before and after NST consultation is depicted in Fig. 1. On POD 25, enteral nutrition was changed from semi-digestive (liquid nutrients with protein as nitrogen source which require digestion for absorption) to semi-solid nutrients (HINE E-GEL®) to alleviate diarrhea. The speed of administration was gradually increased from 80 ml/h to 150 ml/h. Defecation status improved gradually. General condition also became better, and the patient was planned to be transferred to another hospital for further rehabilitation. However, in the early morning on POD 39, the patient suddenly experienced tachypnea and oxygen saturation level declined to 88 %, requiring oxygen support. Suction through both nose and mouth did not show any sputum, and did not improve the situation. Systolic blood pressure also declined to 70 mmHg with pulse rate of 80–90, which required a rapid drip infusion of lactate ringer. Tracheal intubation was then performed to maintain his cardiopulmonary function. During the intubation, there was nothing inside his mouth. After intubation, cough reflex expectorated light brownish sputum with pale yellow gel-form particles measuring 7 × 5 × 3 cm in size (Fig. 2). Pathological examination of the objects revealed artificial contents with fibrous ingredients with a little cell content which was compatible with the ingredients of semi-solid nutrients.

After expectoration of sputum and ventilation support, his pulmonary condition quickly stabilized. Semi-digestive nutrients through the nasogastric tube were resumed on POD 43, and extubation was performed on POD 46. Thereafter, the patient’s general condition was stable and slowly improved. On POD 94, he was transferred to another hospital for further rehabilitation.

## 3 Discussion

Here, an 82-year-old man had tracheal obstruction by aspiration of semi-solid nutrients and subsequent pulmonary failure. This episode was quite rare, but it should be taken into consideration that the reflux of semi-solid nutrients may be more fatal than that of liquid nutrients.

The patient did not have a past medical history of gastrectomy or hiatus hernia which can be anatomical reasons for nutrient reflux. Proton pump inhibitors or calcium, which could change the viscosity of semi-solid nutrients, were not prescribed. Semi-solid nutrients were administered 4 times/day, and the last dose was administered at 9 in the evening and lasted for 3−4 h. The patient was positioned at a 15-degree angle, with the head up during feeding, but after that, the head was partially lowered for sleep, which may have been a reason for reflux.

**Fig. 3:**
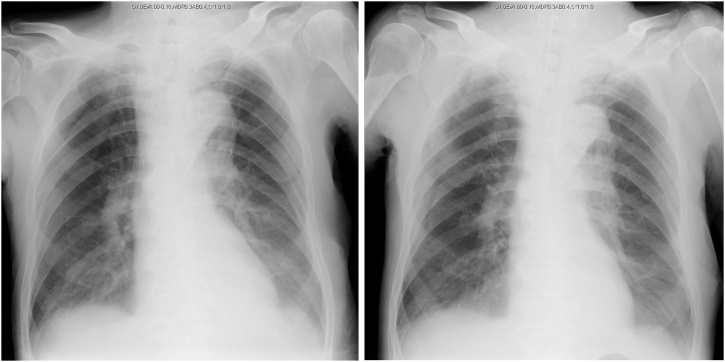
X-rays one day before pulmonary failure (left) and right after the episode (right).

At the time of pulmonary failure, different diagnoses such as pneumonia, pulmonary thrombosis, and pneumothorax were considered. X-rays one day before and right after the episode did not show any findings except slight opacities in the bilateral lower lung areas (Fig. 3). The patient had slight fever, but the inflammatory response was not that high (white blood cell counts: 7.1 × 10^3^ μl; C-reactive protein: 6.71 mg/dL) and improving daily. Sudden onset of pulmonary failure and sudden recovery after intubation following the expectoration of ample sputum, together with gel-form objects, implied that the reflux and aspiration of semi-solid nutrients led to tracheal obstruction.

Benefits of semi-solid nutrients such as reduced diarrhea, gastroesophageal reflux, and aspiration pneumonia were reported in some articles [[1], [2], [3]]. Most published articles focused on semi-solid nutrients through PEG. There is an evident difference between semi-solid nutrients through PEG and through a nasogastric tube. Semi-solid nutrients with high viscosity can be administered through PEG due to its short length, wide diameter, and low intraluminal pressure. The nasogastric tube, on the other hand, is long and narrow, and does not accept semi-solid nutrients with high viscosity which requires high pressure to administer. HINE E-GEL® contains pectin and calcium phosphate which changes from liquid to semi-solid inside the stomach via the chemical reaction of pectin and calcium under acidic conditions. There are not sufficient data on the viscosity of HINE E-GEL® *in vivo*. Uncertainty of its form inside the stomach complicates clinical practice.

In the present case, tracheal obstruction occurred by reflux and aspiration of semi-solid nutrients through the nasogastric tube. Theoretically, similar issues can occur in semi-solid nutrients through PEG, although, to our knowledge, there have been no reports. Semi-solid nutrients as a whole, have clear advantages of reducing gastroesophageal reflux and subsequent aspiration pneumonia, but once reflux and aspiration occur, it can be fatal as was experienced in the present case.

## 4 Conclusion

Although semi-solid nutrients have several benefits and are quite useful in clinical practice, medical staff should be aware that reflux of semi-solid nutrients can occur, and it can be extremely dangerous for the patients.

## Declaration of Competing Interest

All authors declare that there is no conflicts of interest to disclose.

## Sources of funding

There is no funding resource to disclose.

## Ethical approval

The study is exempted from ethical approval in Institutional Review Board of our hospital.

## Consent

Written informed consent was obtained from the patient for publication of this case report.

## Author contribution

Masatoshi Nakagawa and Kaori Sugihara designed and wrote this article. Kiyoshi Isobe, Masafumi Akasu, Kazumine Tsujimoto, Yasuhiro Itsui, Yasuaki Nakajima contributed in data collection and analyses.

## Registration of research studies

This study has not been registered.

## Guarantor

Masatoshi Nakagawa accepts full responsibilities for this work and conduction of this study.

## Provenance and peer review

Not commissioned, externally peer-reviewed.
